# Dynamic Inventory of Intermediate Metabolites of Cyanobacteria in a Diurnal Cycle

**DOI:** 10.1016/j.isci.2020.101704

**Published:** 2020-10-20

**Authors:** Damini Jaiswal, Pramod P. Wangikar

**Affiliations:** 1 Department of Chemical Engineering, Indian Institute of Technology Bombay, Powai, Mumbai 400076, India; 2 DBT-PAN IIT Centre for Bioenergy, Indian Institute of Technology Bombay, Powai, Mumbai 400076, India; 3 Wadhwani Research Centre for Bioengineering, Indian Institute of Technology Bombay, Powai, Mumbai 400076, India

**Keywords:** Microbiology, Omics

## Abstract

Cyanobacteria are gaining importance both as hosts for photoautotrophic production of chemicals and as model systems for studies of diurnal lifestyle. The proteome and transcriptome of cyanobacteria have been closely examined under diurnal growth, whereas the downstream effects on the intermediary metabolism have not received sufficient attention. The present study focuses on identifying the cellular metabolites whose inventories undergo dramatic changes in a fast-growing cyanobacterium, *Synechococcus elongatus* PCC 11801. We identified and quantified 67 polar metabolites, whose inventory changes significantly during diurnal growth, with some metabolites changing by 100-fold. The Calvin-Benson-Bassham cycle intermediates peak at midday to support fast growth. The hitherto unexplored γ-glutamyl peptides act as reservoirs of amino acids. Interestingly, several storage molecules or their precursors accumulate during the dark phase, dispelling the notion that all biosynthetic activity takes place in the light phase. Our results will guide metabolic modeling and strain engineering of cyanobacteria.

## Highlights

• We identify and quantify 67 polar intermediate metabolites in cyanobacteria via LC-MS
• A number of metabolites show large variations during the diurnal cycle
• Intermediates of the CBB cycle peak at midday, coinciding with peak in growth rate
• Gamma-glutamyl dipeptides identified as new storage compounds that peak at dawn

## Graphical Abstract

**Figure:**
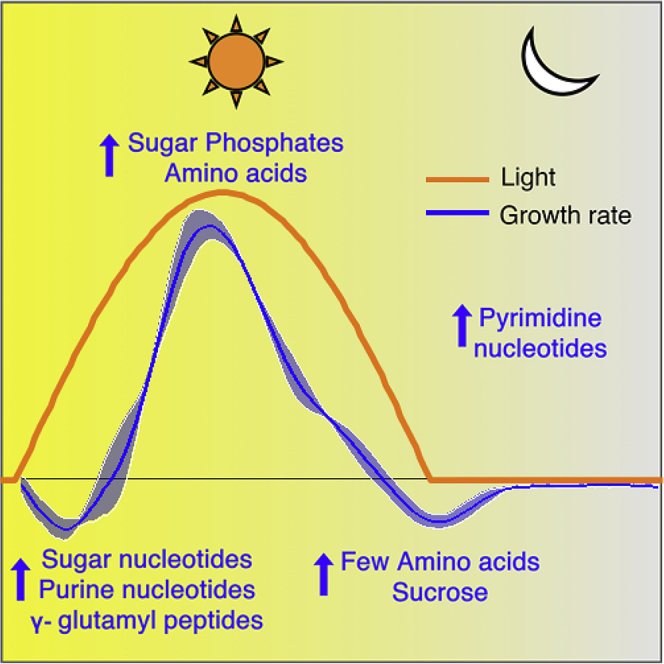

## Introduction

Cyanobacteria are attracting attention as hosts for biotechnological applications due to their efficient photoautotrophy, fast growth, and amenability to genetic modifications (Santos-Merino et al., 2019). Significant advances have been made in the synthetic biology toolbox of cyanobacteria allowing knock-in, knock-out, and transient repression of genes (Knoot et al., 2018; Sengupta et al., 2018). Several studies have demonstrated engineered cyanobacteria that can convert CO_2_ to useful chemicals via an endergonic process driven by light energy (Knoot et al., 2018). However, significant progress is still needed as the product titers have been too low for commercial viability (Knoot et al., 2018). Toward that end, a model-driven and predictive approach has been mooted that may require a detailed and quantitative understanding of cellular metabolism (Jazmin et al., 2017). Thus, metabolomics and fluxomic-based approaches are likely to become an integral part of any strain-engineering pipeline (Fell, 1998; Hendry et al., 2017; Jang et al., 2018; Mathew and Padmanaban, 2013; Wiechert and Nöh, 2013). These approaches can be broadly categorized as follows with their implications in model-driven biotechnology: (1) detection and identification of a large number of intracellular and extracellular metabolites (n > 100), which may aid in refining the genome-scale metabolic reconstruction (Narainsamy et al., 2013; Thiele and Palsson, 2010); (2) absolute or relative quantification of a moderate number of metabolites (n = 50–100) (Schwarz et al., 2013), that can help in solving metabolic models that explicitly require metabolite pool sizes; and (3) quantification of isotopic ^13^C enrichment in a limited number of metabolites of the central carbon network (n = 10–25) with emphasis on model-driven estimation of reaction rates of the key central pathways (Hendry et al., 2017; Jaiswal et al., 2018a; Young et al., 2011). The third approach also requires the knowledge of metabolite pool sizes, although they are typically estimated by fitting a network model to the ^13^C enrichment profiles and not measured experimentally due to the challenges associated with absolute quantification (Young, 2014; Young et al., 2011).

Dempo and co-workers have estimated absolute pool sizes of 83 cellular metabolites in a first-of-its-kind study in cyanobacteria (Dempo et al., 2014). These 83 metabolites together account for ~6% w/w of typical cyanobacterial biomass, with a majority of the metabolites being present at levels below 0.2 μmole/g dry cell weight, reinforcing the notion that the intermediate metabolites are present in low abundance. Glutamate was found to be the most abundant metabolite with a pool size of ~210 μmole/g dry cell weight in *Synechococystis* sp. PCC 6803. Other significant metabolomics studies with cyanobacteria have explored metabolic changes in response to CO_2_ acclimatization (Eisenhut et al., 2008; Schwarz et al., 2011), nitrogen starvation (Qian et al., 2018), glycogen synthesis impairment (Cano et al., 2018; Gründel et al., 2012), mixotrophic growth (Takahashi et al., 2008), and other stresses (Hasunuma et al., 2018; Miranda et al., 2013; Wang et al., 2014). While the technological advancements in liquid chromatography coupled with tandem mass spectrometry (LC-MS/MS) have significantly expanded the repertoire of metabolites that can be analyzed, challenges still remain in identifying and quantifying a large number of metabolites (Baran et al., 2010, 2011, 2013; Dempo et al., 2014; Jaiswal and Wangikar, 2020; Jaiswal et al., 2020a; Narainsamy et al., 2013). Furthermore, the majority of the reports have used cultures grown under continuous light (CL), thus ignoring the effect of diurnal lighting, which the strain will be subjected to during the eventual outdoor growth and commercial applications.

Cyanobacteria are also being widely used as models for studying the circadian clock and diurnal lifestyle. The internal circadian clock of cyanobacteria can influence the metabolic rhythms under both diurnal and CL conditions (Diamond et al., 2015; Welkie et al., 2018). The circadian clock of cyanobacteria confers fitness to the organism, possibly by anticipating and preparing for the next phase during the diurnal growth (Welkie et al., 2018). Cyanobacteria perform photosynthesis and produce biomass components and storage molecules during the day, which are broken down in the night to draw energy for sustenance. Apart from the circadian control over the metabolism, the light-dark cycle can also drive metabolic rhythm independently of the clock (Diamond et al., 2015; Pattanayak et al., 2014; Saha et al., 2016; Sengupta and Wangikar, 2020). To exemplify, the oscillations in glycogen synthesis and degradation have been reported in the wild-type as well as Δ*kaiC* strains when grown in diurnal cycle (Diamond et al., 2015; Suzuki et al., 2007). Note that the KaiC protein is well known for its central role in the cyanobacterial circadian clock and undergoes a cycle of phosphorylation and dephosphorylation over the period of 24 h. While detailed studies have been performed on how the transcriptome and proteome oscillate in a diurnal cycle, there are limited studies at the metabolome level (Diamond et al., 2015; Fleming and O'Shea, 2018; Guo et al., 2014; Iijima et al., 2015; Krishnakumar et al., 2013). Sarkar et al. have accounted for inventorying and consumption of metabolites in a diurnal flux balance model of *Synechocystis* sp. PCC 6803 by creating time point models spanning the light-dark cycle (Sarkar et al., 2019). More recently, Werner et al. have reported the metabolite levels of that strain in a sinusoidal light-dark (sine LD) cycle and show differential accumulation of metabolites in certain phases of the day (Werner et al., 2019). This study, however, did not include some key intermediates of the Calvin-Benson-Bassham (CBB) cycle, such as ribulose-1,5-bisphosphate (RuBP), 3-phosphoglyceric acid (3PGA), sedoheptulose-1,7-bisphosphate (SBP), and sedoheptulose-7-phosphate (S7P) that are involved in the reactions mediated by carbon flux-controlling enzymes (Janasch et al., 2018; Liang and Lindblad, 2016). Thus, there is a need to systematically explore the dynamic inventory of cyanobacterial metabolites during the diurnal cycle.

Here we present an LC-MS/MS-based, large-scale metabolite profiling of the fast-growing cyanobacterium *Synechococcus elongatus* PCC 11801 (henceforth *S. elongatus* PCC 11801) in a diurnal sine LD cycle. *S. elongatus* PCC 11801 is a promising candidate for biotechnological applications owing to its faster growth, tolerance to high light and temperature, and genetic amenability (Jaiswal et al., 2018b, 2020b; Sengupta et al., 2020a, 2020b). We find dramatic intra-day changes in the inventory of sugar bisphosphates, sugar phosphates, nucleotides, sugar nucleotides, organic acids, amino acids, and hitherto unexplored γ-glutamyl dipeptides. Importantly, certain intermediate metabolites were found to accumulate significantly during the night, suggesting that their biosynthesis is not confined to the dawn-to-dusk period. Our results will have significant implications in rational strain engineering strategies.

## Results

### LC-MS/MS Method for Cyanobacterial Metabolomics

**Figure 1:**
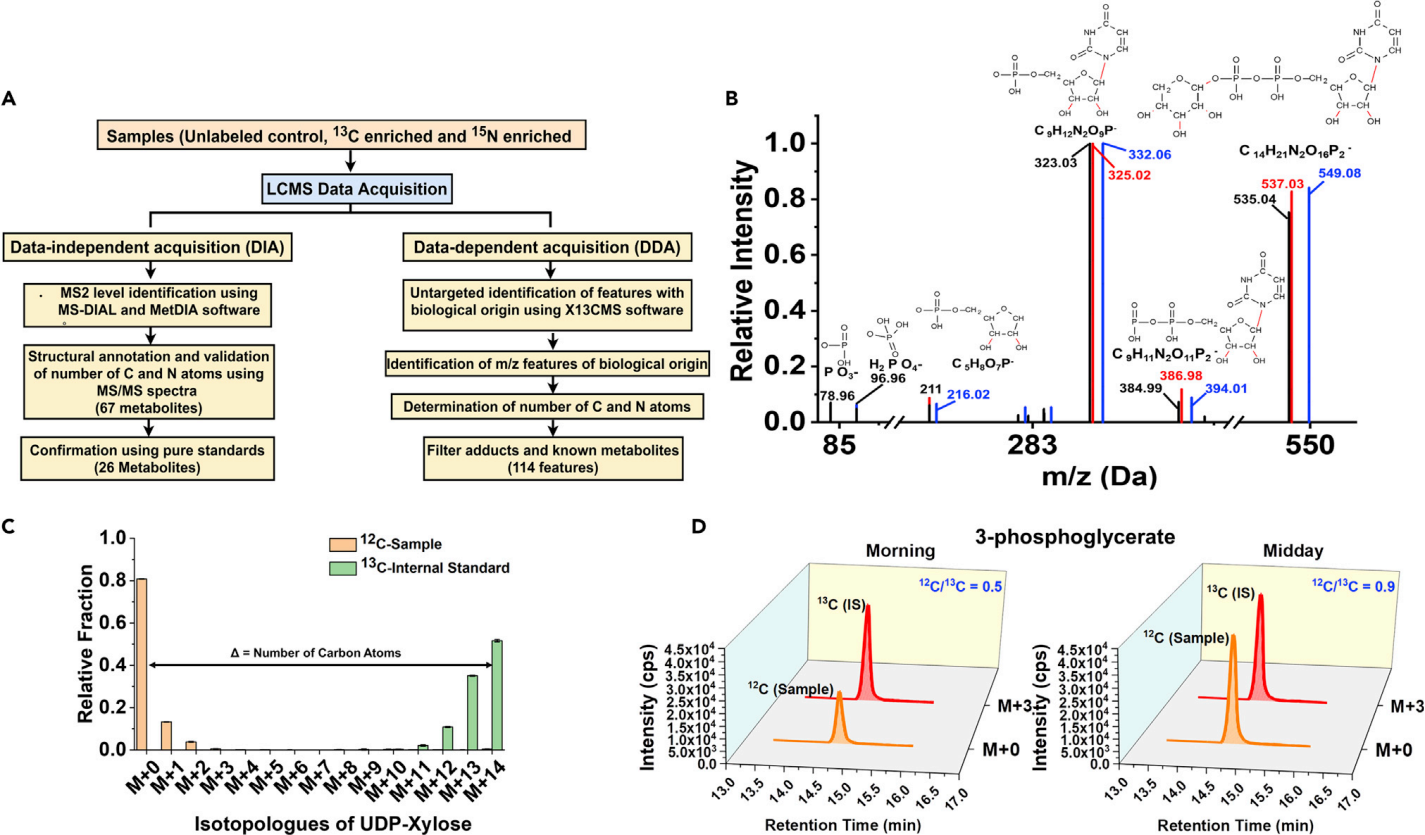
LC-MS/MS Method for the Detection, Identification, and Relative Quantification of Cyanobacterial Metabolites (A–D) (A) Overall methodology: Tandem mass spectrometry (MS2) data were acquired in data-dependent (DDA) and data-independent SWATH modes and the latter used for metabolite identification (see Transparent Methods and Tables S2–S4, and S5 for further details). The carbon and nitrogen composition of the metabolites and their fragments was confirmed from the mass shifts observed in the ^13^C- or ^15^N-labeled metabolite extracts. X^13^CMS software was used to detect isotopic enrichment in an untargeted manner. (B) MS2 spectra of UDP-xylose in negative ion mode, a representative metabolite identified by MS-DIAL. The black, blue, and red bars represent the mass spectra of the monoisotopic peaks from the control, ^13^C-, and ^15^N-enriched samples, respectively. (C) The mass isotopologue distribution (MID) of the control and ^13^C-enriched samples as obtained from X^13^CMS, exemplified with UDP-xylose. (D) Quantification of relative metabolite levels using isotopic area ratio method (^12^C/^13^C) exemplified for 3-phosphoglyceric acid (3PGA) in two conditions. An equal amount of ^13^C-enriched metabolite extract of *S. elongatus* PCC 11801 was added in all samples, thus affording a retention time-matched and mass-shifted internal standard for each metabolite.

The main analytical objective was to detect, identify, and quantify a large number of cellular metabolites of cyanobacteria. It is now well known that MS detectors can be used for quantification with the use of appropriate internal standards (IS) (Qiu et al., 2016; Stupp et al., 2013). We used the metabolite extract of a ^13^C-labeled cyanobacterial biomass as the IS, which provides a retention time-matched but mass-shifted peak that serves as IS for each metabolite, including the unidentified m/z features (Dempo et al., 2014; Qiu et al., 2016). This poses an additional challenge in analytical method development as the mixture of the sample and IS results in a many-fold increase in the number of m/z features and, in turn, an increased frequency of coelution of isobars. As our interest was in the intermediate metabolites, which are typically charged and polar, we initially explored the hydrophilic interaction liquid chromatography and ion-pairing-based liquid chromatography methods for separation (Jaiswal et al., 2020a). Although each method has its advantages, we chose the latter primarily because it detects a larger number of metabolites, yields better quality peaks, and provides baseline separation for isomers such as glucose-6-phosphate (G6P) and fructose-6-phosphate (F6P) (Jaiswal et al., 2020a; Lu et al., 2010; Luo et al., 2007; Mccloskey and Gangoiti, 2015; Qian et al., 2004). The instrument was operated in negative ion mode, and further details of instrument parameters and methods could be found in Supplemental Information (see Transparent Methods and Tables S1–S4). The MS data were collected in an untargeted manner and analyzed in a targeted way, which involves the following steps: (1) annotation of peaks by matching the tandem mass spectra (MS2) with those of pure standards or with a database using the tools MS-DIAL (Tsugawa et al., 2015) and MetDIA (Li et al., 2016) (Figure 1A and Table S5); (2) confirmation that the peaks are of biological origin by verifying that the mass shifts for ^13^C or ^15^N isotopically labeled biomass samples correspond to the carbon and nitrogen compositions (Figures 1B and 1C); and (3) quantification of the areas under the curve for the ^12^C and ^13^C monoisotopic peaks to obtain the isotopic area ratios of sample/IS for each metabolite (Figure 1D) (Qiu et al., 2016; Stupp et al., 2013). Note that steps (2) and (3) can be performed for the unidentified m/z (mass to charge ratio) features to obtain their C/N composition and isotopic area ratios with the possibility of annotating them at a later stage.

### Diurnal Growth of Cyanobacteria, Sampling Strategy, and Data Analysis

**Figure 2:**
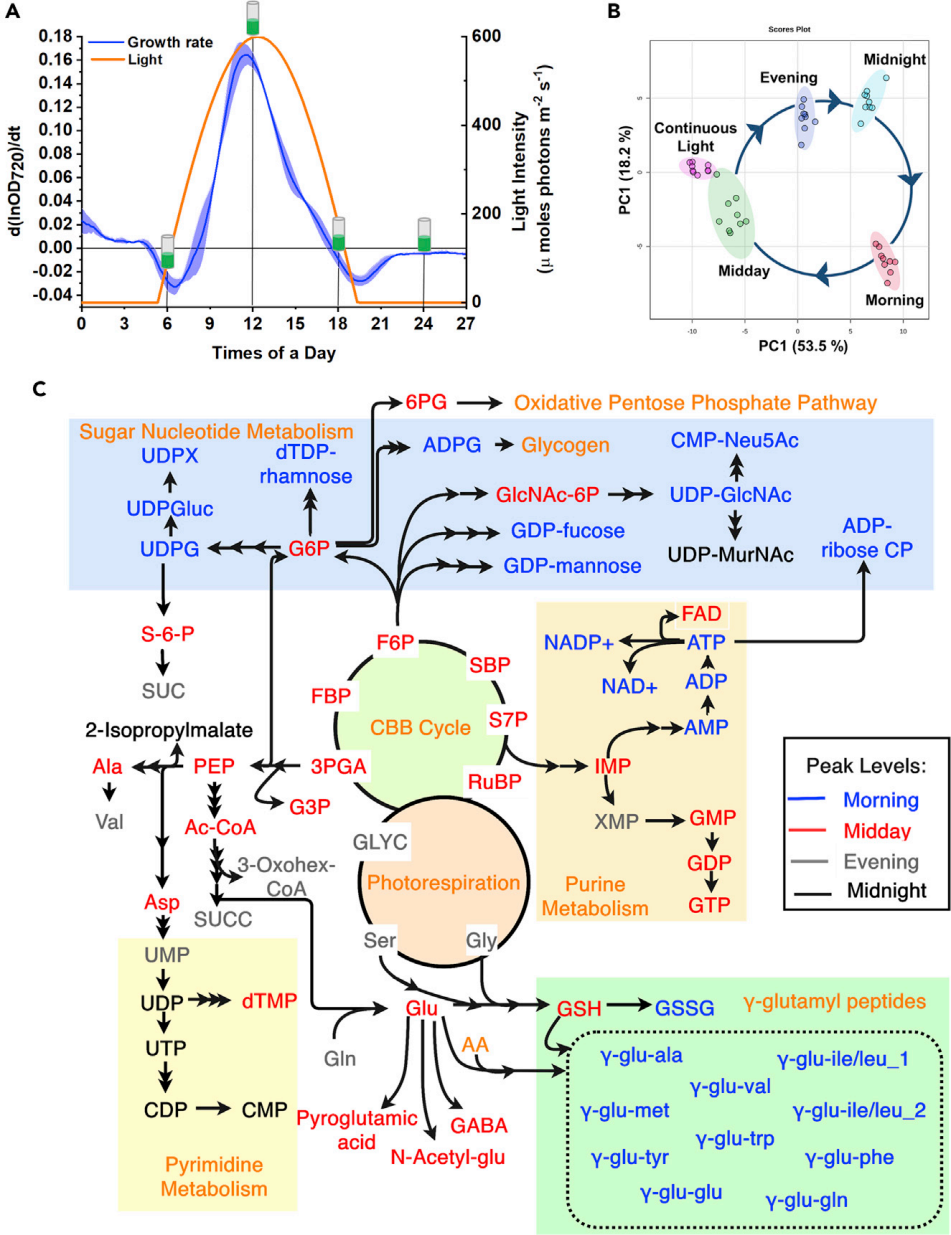
Diurnal Growth of *S. elongatus* PCC 11801, Sampling Strategy, and Data Analysis (A and B) (A) The light profile and instantaneous specific growth rate (μ) in the second diurnal cycle (day 2). Samples for metabolomics analysis were collected at 6:00, 12:00, 18:00, and 24:00 h and referred to as morning (M), midday (MD), evening (E), and midnight (MN), (B) Principal-component analysis (PCA) score plot based on the relative levels of the 67 annotated metabolites for the replicates under the five conditions. The area with 95% confidence interval of each group is highlighted. (C) Metabolic pathway with peaking times of the metabolites in a diurnal cycle indicated by the color of the font; M (blue), MD (red), E (gray), and MN (black). Refer to Table S5 for the full form of abbreviations used.

The key objective was to identify the metabolites that change significantly, their peaking times, and fold changes in their inventories in a diurnal cycle. To mimic the natural lighting of its native habitat (Jaiswal et al., 2018b), *S. elongatus* PCC 11801 was grown in diurnal cycles comprising 14 h of sinusoidal light with peak light intensity of 600 μmole photons.m^−2^.s^−1^ and 10 h of darkness (Figure 2A). The OD_720nm_ profile has been shown for four diurnal cycles (Figure S1), which was then used to compute the instantaneous specific growth rate (μ). The profile of μ roughly follows the light intensity pattern and reaches its peak value of 0.16 ± 0.01 h^−1^ at midday (Figure 2A). This compares well with that obtained under CL of 600 μmole photons.m^−2^.s^−1^ (Jaiswal et al., 2018b). Interestingly, the negative growth rate of OD_720_ was observed for 1–2 h just after sunrise and just before sunset. This pattern was found to be highly reproducible not only in biological replicates and consecutive diurnal cycles but also for growth experiments with peak light intensity of 400 μmole photons.m^−2^.s^−1^ (Figures S2–S4). Although further investigations are needed to pinpoint the cause of such negative growth rate at dusk and dawn, a previous study on cyanobacteria suggests that the nighttime decline in OD_720_ may be due to loss of biomass rather than due to a decrease in the cell number (Werner et al., 2019). In view of the observed diurnal growth pattern, the metabolome was profiled at 6:00 (morning), 12:00 (midday), 18:00 (evening), and 24:00 (midnight), in the second diurnal cycle after culture inoculation (Figure 2A). Culture grown under CL constitutes the fifth data point.

Overall, isotopic area ratios were quantified for 67 annotated metabolites (Table S6) and 114 unannotated m/z features under five conditions. One-way ANOVA showed that 65 metabolites vary significantly between any two conditions at a fold change > 1.5 at p value < 0.05. Principal-component analysis (PCA) was performed to assess reproducibility among the replicates and the variation between the five conditions on the metabolome landscape (Figure 2B). Broadly, the replicates were tightly clustered, whereas the five conditions were well separated from each other. The samples from the CL condition were closest to the diurnal cycle's midday condition, although these two conditions form two separate clusters pointing toward some key differences. The midday and midnight samples were in the opposite quadrants of the scores plot, suggesting that they differ the most in their metabolomes. Interestingly, the four time points of the diurnal cycle occupy places on the scores plot that appear to result from a cyclic process (Figure 2B). This also suggests that distinct metabolic events characterize each phase of the diurnal cycle with the cell anticipating and getting ready for the next phase. Furthermore, the detected metabolites have been shown in the context of the pathways that they participate in with color codes depicting their peaking times (Figure 2C). The annotated metabolites included the intermediates of glycolysis, CBB cycle, tricarboxylic acid cycle, biosynthetic pathways, nucleotides, sugar nucleotides, amino acids, and γ-glutamyl dipeptides. It was of interest to analyze how metabolite levels change during the diurnal cycle. Clearly, several metabolites of a pathway appear to peak in concert as exemplified by those of the CBB cycle that peak at midday.

### Intermediates of the CBB Cycle and Other Central Carbon Pathways Peak at Midday

**Figure 3:**
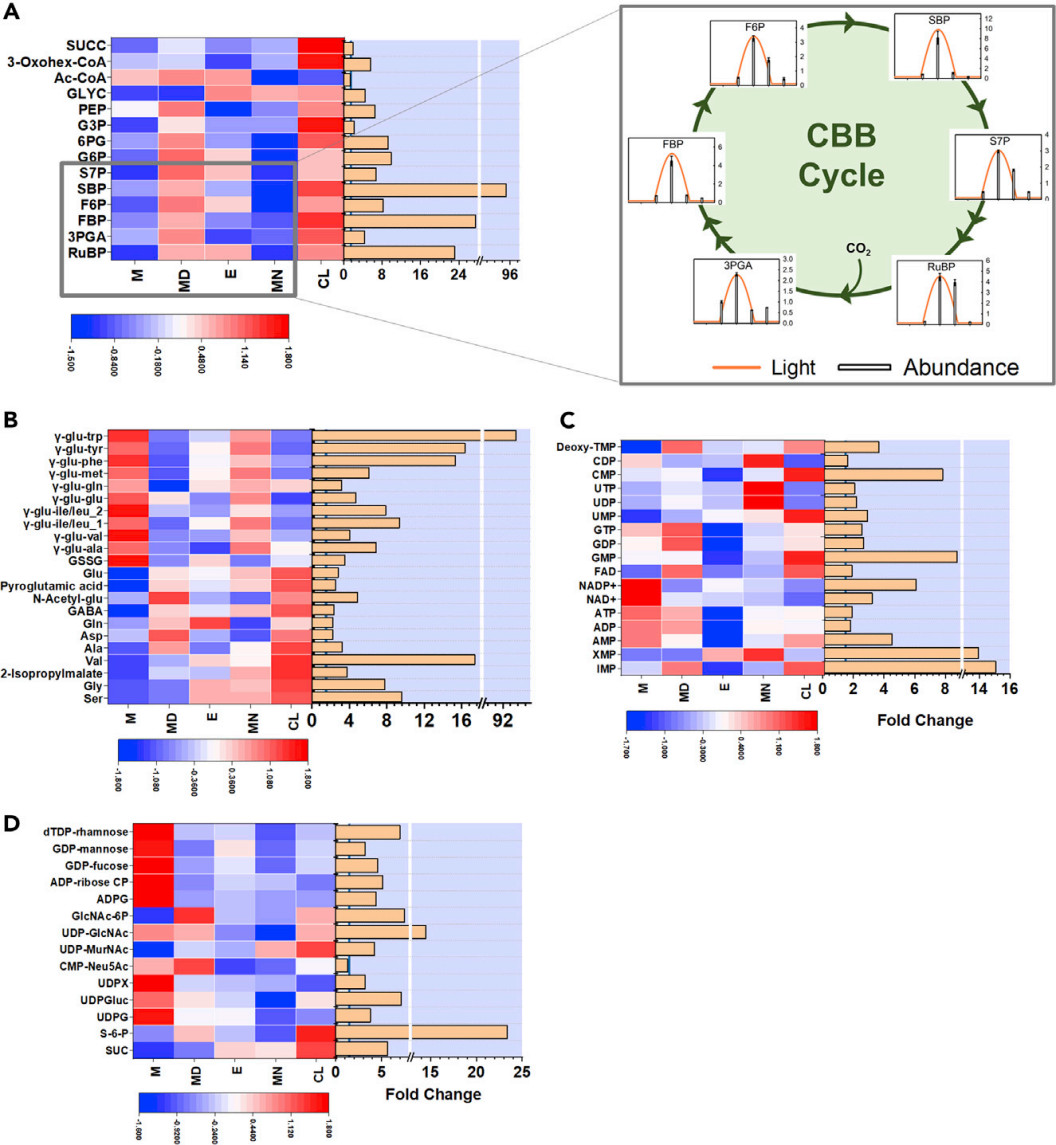
Diurnal variation in metabolite levels Heatmaps, grouped by metabolic pathways, show variation in the levels of intermediate metabolites at different times in a diurnal cycle and under continuous light. (A–D) (A) Intermediates of the CBB cycle and other central carbon pathways, (B) amino acids and γ-glutamyl peptides, (C) nucleotides, and (D) sugar nucleotides and related metabolites. The data were log_2_ transformed, averaged over the replicates, and auto-scaled. The inset of (A) shows values normalized by geometric mean across the five conditions. The fold changes (maximum/minimum) in metabolite levels across the five conditions are shown as bar graphs. All the metabolites showed fold change ≥1.5 at p value ≤ 0.05 using ANOVA barring acetyl coenzyme A (Ac-CoA) and CMP-N-acetylneuraminate (CMP-Neu5Ac). See also Tables S5 and S6.

We visualized the overall data as heatmaps with metabolites grouped according to pathways (Figures 3A–3D). Although the heatmap shows patterns with auto-scaled data, the magnitude of change for each metabolite is viewed as bar graphs and placed adjacent to the heatmap. Among the central carbon metabolites, SBP, fructose-1,6-bisphosphate (FBP), and RuBP show the largest fold change with the highest and lowest levels at midday and midnight, respectively (Figure 3A). These are the substrates for the three irreversible steps of the CBB cycle, and a substantial fold change in their levels in unison suggests their role in regulatory mechanisms. Other intermediates of the CBB cycle, the glycolytic and pentose phosphate pathways, also peak at midday (Figure 3A, see the inset). Compared with midday, lower levels are observed in morning and evening, where the instantaneous growth rate is much lower (Figure 2A), with the minimum levels observed at midnight. Although the CBB cycle intermediates peak in concert at midday, the extent of depletion by evening varies for the different metabolites. For example, the levels of the metabolites like F6P, S7P, and RuBP show comparatively lower fold decline from midday to evening. As gene expression data are not available for the strain *S. elongatus* PCC 11801, we made an attempt to correlate our results with the transcriptomics data under diurnal and circadian conditions for other cyanobacteria such as *S. elongatus* PCC 7942 and *Cyanothece* sp. ATCC 11801 (Stöckel et al., 2008; Vijayan et al., 2009). Indeed, the expression of the bifunctional fructose-1,6/sedoheptulose-1,7-bisphosphatase (FBP/SBPase) peaks at subjective dusk (Vijayan et al., 2009), correlating with the rapid decline in the levels of its substrates SBP and FBP by dusk (Figure 3A, inset). Furthermore, dusk-peaking of the *tal* (transaldolase) gene may have a role in avoiding a similar rapid decline in the levels of F6P. Moreover, *tal* gene has been found to be essential for growth under diurnal cycle (Welkie et al., 2018). We hypothesize that the levels of the CBB cycle and other central metabolites rise in a coordinated manner around midday to support high enzymatic reaction rates of the central pathways and, in turn, a high growth rate. Note that these metabolites show high levels under CL as well, and the genes involved in their formation have been considered essential under CL condition (Rubin et al., 2015).

### γ-Glutamyl Peptides Are Potential Reservoirs of Amino Acids

Although the chromatography method detected 19 amino acid standards, only seven were detected in the cyanobacterial samples (Jaiswal et al., 2020a). The amino acids glu, asp, gln, gly, ala, ser, and val and the derivatives, pyroglutamic acid and N-acetyl glutamic acid (N-Acetyl-glu) showed satisfactory peaks in the samples and showed higher abundance during midday. In fact, they show trends that are similar to the central carbon metabolites. However, the hydrophobic amino acids trp, tyr, phe, leu, ile, and met could not be detected in the samples. Interestingly, γ-glutamyl peptides of these amino acids showed prominent peaks in our chromatography with significant accumulation through the night but rapid depletion by midday. Large fold changes were observed for majority of these peptides (Figure 3B). It is known that some of the hydrophobic amino acids have better solubility and stability in peptide forms (Baran et al., 2011). In cases where both the free amino acid and the respective dipeptide are detected, the dipeptides show the largest pool size in the morning, whereas the free amino acid shows the largest pool size at midday (Figure 3B). Based on these results, we hypothesize that the cell continues to synthesize these amino acids in the dark phase and sequesters them as dipeptides to be utilized during the light phase when a fast growth rate is to be achieved. Our hypothesis is consistent with reports on another nitrogen reservoir in cyanobacteria, cyanophycin, that accumulates during unbalanced growth conditions (Flores et al., 2019). Notably, cyanophycin also accumulates during the dark condition in diazotrophic cyanobacteria, and the genes for its synthesis have been found to be maximally expressed around dusk (Li et al., 2001; Saha et al., 2016; Stöckel et al., 2008). Moreover, the genes for biosynthesis of aforesaid hydrophobic amino acids were also found to peak at dusk in *Cyanothece* sp. ATCC 51142 (Stöckel et al., 2008). Also, note that the amino acids trp, tyr, phe, leu, and ile, whose dipeptides show accumulation and, in turn, significant biosynthetic activity during the night, require multi-step synthetic pathways starting from their precursors drawn from the central carbon pathway. Most amino acids peaked at midday and the γ-glutamyl peptides peaked at morning, whereas the amino acids gly, gln, and ser appeared to be exceptions with peaks in the evening. Furthermore, the abundance in CL correlated with that at midday for most compounds except for gly and ser (Figure 3B).

### Sugar Nucleotides and Purine Nucleotides Accumulate in the Morning, whereas Uridine Nucleotides at Midnight

Several of the nucleotides, especially the abundant ones such as ATP and ADP, showed relatively small fold changes in the diurnal cycle (Figure 3C). On the other hand, nucleotide monophosphates such as CMP, GMP, IMP, and XMP showed larger fold changes. Nucleotides are the precursors for the biosynthesis of DNA, RNA, cofactors, and coenzymes. Nucleotides such as ATP and GTP are also energy carriers and play an essential role in driving reactions that are thermodynamically unfavorable (Asplund-Samuelsson et al., 2018). They are also components of activated biosynthetic intermediates like UDP-glucose and ADP-glucose. Therefore the differential accumulation patterns of nucleotides would be of interest in metabolic modeling and strain engineering studies. Interestingly, the purine and pyrimidine nucleotides show different accumulation patterns (Figure 3C).

IMP, the first nucleotide precursor for the synthesis of purine nucleotides that is synthesized from R5P, has an accumulation pattern similar to sugar phosphates and peaks during midday (Figures 2C and 3A). IMP then forms adenylosuccinate and XMP via the reactions catalyzed by adenylosuccinate synthetase and IMP dehydrogenase, respectively. Both these enzymes are regulated through feedback inhibition by their respective downstream products AMP and GMP (Pimkin and Markham, 2008). The data for XMP and GMP are consistent with the reported feedback inhibition as the XMP levels are lower in the conditions where GMP levels are higher (morning and midday, Figure 2D). All the purine nucleotides showed accumulation during the morning and a sharp decline toward the evening. These results suggest that the cells accumulate these nucleotides in preparation for photosynthesis, DNA replication, and cell growth that majorly occurs during the day. The genes involved in nucleotide biosynthesis and these cellular activities also peak during the day (Saha et al., 2016). Furthermore, the decline in the levels of purine nucleotides during the evening is greater for nucleoside monophosphates, AMP, and GMP (Figure 3C). The levels of purine nucleotides were restored in the dark, probably through the glycogen catabolism and shunting of glucose to oxidative pentose phosphate pathway (Saha et al., 2016; Stöckel et al., 2008; Welkie et al., 2019). We observed significant accumulation of the oxidized cofactors NAD+ and NADP+ at dawn. It has been reported previously that the levels of these oxidized cofactors increase, whereas their reduced protonated equivalents (NADH and NADPH) deplete in the dark condition (Iijima et al., 2015). We hypothesize that a similar phenomenon may be responsible for the accumulation of the oxidized cofactors at dawn, although we could not detect and quantify the reduced cofactors NADH and NADPH in our chromatography method. Unlike the purine nucleotides, the pyrimidine nucleotides, especially uridine nucleotides, show the highest abundance at midnight (Figure 3C). Accumulation of uridine nucleotides during the night has not been reported previously. These results suggest that the cell may have distinct metabolic requirements of purine and pyrimidine nucleotides apart from their common role in DNA and RNA synthesis.

The sugar nucleotides are activated forms of monomeric sugar, the anomeric carbon of which is attached to a nucleotide through a phosphate ester linkage. They are donors for monomeric sugar residues in glycosylation reactions forming polysaccharides. Thus, they are key intermediates in the synthesis of the cell wall and storage compounds such as glycogen and sucrose. We find that majority of the sugar nucleotides peak in the morning, except for UDP-MurNAc that peaked at midnight (Figure 3D). We hypothesize that the levels of sugar nucleotides rise in the morning as a preparatory step to support fast growth rate during the day. The genes involved in sugar nucleotide metabolism were maximally expressed at subjective dawn in *S. elongatus* PCC 7942 and thus correlated with our data (Vijayan et al., 2009). The midday and the evening are marked by lower levels of these sugar nucleotides, possibly due to their utilization in the synthesis of polysaccharides. UDP-MurNAc is an important activated intermediate of cell wall biosynthesis and requires uridine nucleotide in its synthesis. We hypothesize that the peaking of UDP-MurNAc at midnight may be related to the concomitant peaking of uridine nucleotides (Figure 3C).

### Comparison of Metabolite Levels between Midday and Continuous Light

**Figure 4:**
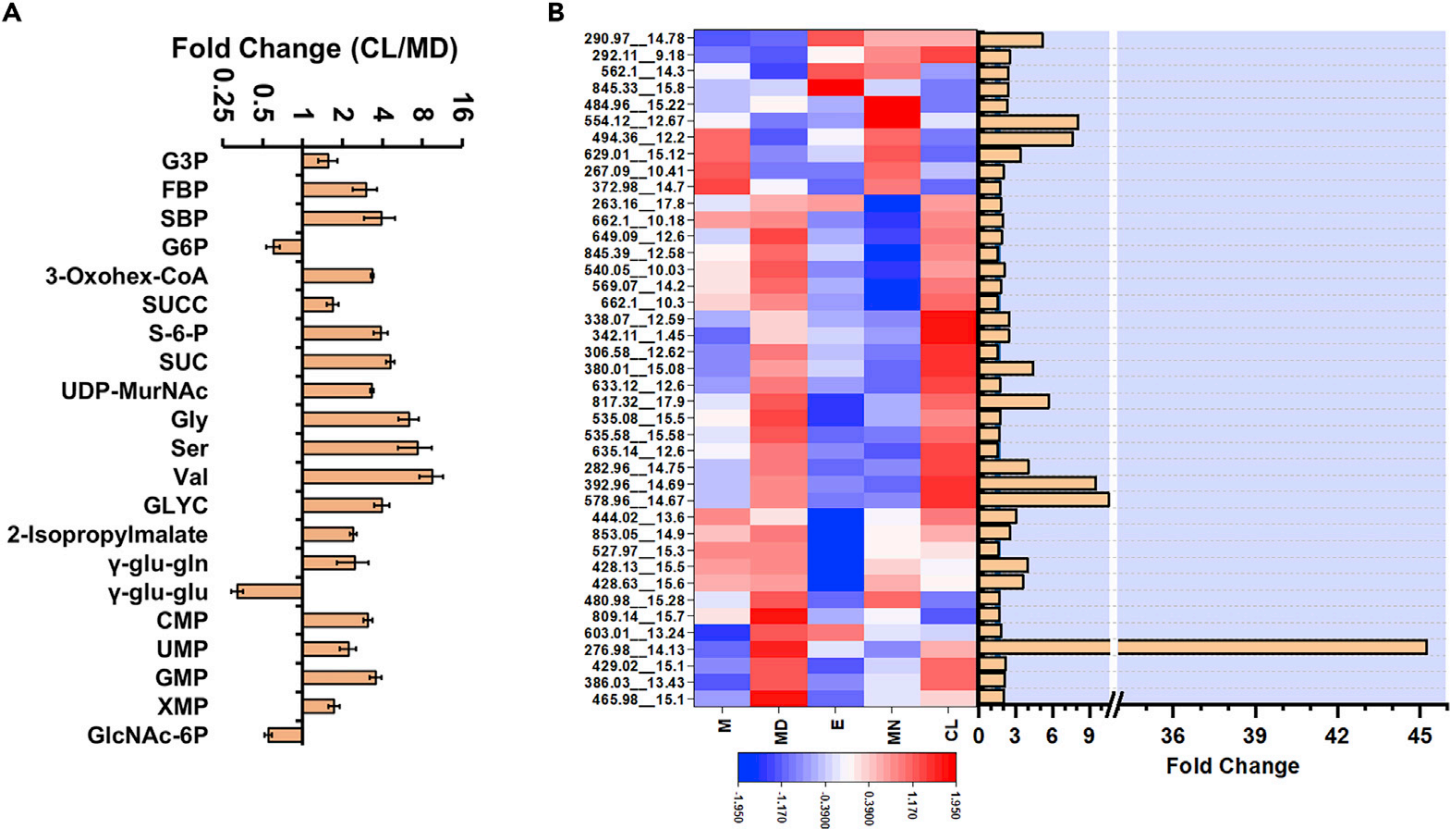
Comparison of Metabolite Levels between Midday and Continuous Light and an Overview of the Diurnal Changes in Unannotated m/z Features (A) The metabolites with a fold change ≥1.5 at p value ≤ 0.05 between midday (MD) and continuous light (CL) using Student's t test. (B) The heat map shows accumulation pattern of unannotated m/z features while the adjacent bar graph shows their fold changes (maximum/minimum) across the five conditions. Of the 114 features identified as those of biological origin, data are presented for 41 features that satisfy the conjunctive criteria of fold change ≥1.5 at p value ≤0.05 and average intra-sample coefficient of variation (CV) ≤ 0.3. See also Tables S7 and S8.

Laboratory research on cyanobacteria is typically performed under an artificial condition of CL, whereas the eventual application may involve outdoor cultivation under sunlight that follows diurnal cycles. Thus, it was of interest to compare the metabolite profiles between these two conditions. A direct comparison was performed between MD and CL conditions, both of which have identical light intensities (Figure 4A). Only 21 of the 67 metabolites showed a significant difference between these two conditions (fold change >1.5, p value < 0.05). Similar to the midday condition, the intermediates of the central carbon pathway maintain high abundance under CL, possibly to sustain a high growth rate. Among the ones that show the difference, several are midday-peaking that show even greater abundance in CL. Examples of this category include SBP and FBP, whose abundance is known to be dependent on light intensity with the CL condition enabling higher accumulation. On the other hand, metabolites that show the lowest abundance at midday show similar abundance under CL. This includes the γ-glutamyl peptides that act as reservoirs of amino acids under the diurnal cycle, suggesting that the need for sequestration of the latter may become redundant under CL by balancing of their synthesis and consumption. Interestingly, gly, ser, and glyceric acid show significant accumulation under CL. These metabolites are involved in the pathways responsible for mitigating light stress, and thus their levels are expected to vary significantly between the diurnal and CL conditions. Notably, the differences in fitness of metabolic genes between CL and light-dark conditions were found to be linked to the pathways that alleviate light stress (Welkie et al., 2018). The nucleotide monophosphates such as GMP, UMP, and CMP show significant accumulation under CL compared with midday.

### Untargeted Analysis Reveals Diurnal Changes in Unannotated m/z Features

As the LC-MS data are collected in an untargeted manner, they contain information on several as-yet unannotated metabolites, typically termed as the m/z features. Although the use of a pure standard reference compound remains the gold standard for accurate peak annotation, these are unavailable in many cases. Moreover, the presence of a large proportion of background noise in the form of adducts, in-source fragments in combination with matrix effects, poses limitations for LC-ESI-HRMS (liquid chromatography-electrospray ionization-high-resolution MS)-based metabolomics (Bueschl et al., 2014). Stable isotope labeling is a powerful technique that alleviates most of these limitations in untargeted metabolomics and has been widely used to determine the chemical composition of compounds (Baran et al., 2010; Bueschl et al., 2014; Giavalisco et al., 2011). Apart from the annotated metabolites, we confirmed 114 m/z features to be of biological origin and estimated their carbon and nitrogen composition based on the mass shifts in ^13^C- and ^15^N-labeled samples, respectively. The multimer ion adducts of the annotated compounds such as 3PGA and G6P were not included in this list (Prasannan et al., 2019). A key aspect of our approach is that a retention time-matched, mass-shifted IS is available in the ^13^C-labeled metabolite extract even for the unannotated m/z features. Apart from the criterion of fold change > 1.5 and p value < 0.05, an additional filter was applied to select m/z features that show the intra-sample coefficient of variation (CV) <0.3, resulting in 41 m/z features (refer to Table S7 for isotopic area ratios). Figure 4B shows an overview of how the inventory of the unannotated m/z features changes in the diurnal cycle and CL. A number of m/z features showed >10-fold variation in the diurnal cycle (Figure 4B). A large number of m/z features peak at midday and CL conditions, thus indicating that their synthesis might be regulated by light availability. Furthermore, the abundance levels were found to be similar under the midday and CL conditions for majority of the metabolites. As the next step, it would be of interest to identify these m/z features.

## Discussion

We show statistically significant changes in the levels of 65 metabolites and 41 unannotated m/z features during the diurnal growth of the fast-growing *S. elongatus* PCC 11801. A number of factors determine if a given metabolite will meet the significance criteria, including its abundance, peak quality, signal-to-noise ratio, presence of co-eluting isobaric compounds, and the resolution and sensitivity of the mass detector. We have ascertained that all the unannotated m/z features correspond to compounds of biological origin based on the mass shifts in the ^13^C- or ^15^N-labeled samples. Furthermore, their C and N compositions have been obtained, paving the way for their identification in future studies (Table S8). Some metabolites show inventory buildup during the day and consumption during the night, whereas others show peaking at midnight or early morning. Interestingly, we find large fold changes in intermediate metabolites that are not considered to be storage molecules.

We hypothesize that metabolite levels can change in a diurnal cycle to serve two distinct purposes: (1) synthesize and build inventory in one phase for consumption in another phase and (2) increased levels to support higher rates of enzymatic reactions in certain phase(s). It is well-known that the storage compounds such as glycogen fall in the first category with synthesis during the day and consumption during the dark (Diamond et al., 2015; Pattanayak et al., 2014). Our results suggest that the γ-glutamyl peptides are also examples of this category but are synthesized during the night for consumption during the day. The accumulation pattern of γ-glutamyl peptides suggests that these may play the role of a nitrogen reservoir during unbalanced growth conditions, very similar to that played by cyanophycin in diazotrophic cyanobacteria. Interestingly, *S. elongatus* PCC 11801, and its closest model cyanobacterium, *S. elongatus* PCC 7942, both lack genes for the synthesis of cyanophycin (Li et al., 2001). Thus, in the absence of other known nitrogen reservoirs, the role of γ-glutamyl peptides as potential nitrogen reservoirs needs to be explored further. Interestingly, the genes for certain hydrophobic and aromatic amino acids are reported to be maximally expressed during the dark phase in diazotrophic cyanobacteria (Stöckel et al., 2008). Although the prior results of gene expression for other cyanobacteria help interpret the present results to some extent, a detailed gene expression study of *S. elongatus* PCC 11801 under diurnal growth would be needed to fully understand the pathways that may be active during the dark phase in this non-nitrogen fixing cyanobacteria.

The concept of phase-wise inventory building and utilization has been explored in recent modeling studies (Reimers et al., 2017; Sarkar et al., 2019). Majority of such models are based on FBA and do not explicitly deal with metabolite levels or enzyme kinetics. For example, Sarkar et al. (2019) have modeled the net accumulation and consumption of many metabolites primarily to address the requirement that they need to be synthesized and utilized in specific phases. However, the coordinated rise in the levels of CBB cycle intermediates at midday (Figure 3A) cannot be justified as a simple inventory building exercise for the following reasons: (1) the phosphorylated intermediates of the CBB cycle constitute a small fraction of the total biomass (Dempo et al., 2014) and are not known to play the role of storage molecules, (2) the metabolite levels decline significantly by evening thus ruling out their role in providing energy for the dark phase, and (3) the culture shows a dramatic increase in the instantaneous growth rate around midday, concomitant with the rise in the metabolite levels. The transcriptomics and proteomics studies have shown that the levels of enzymes of the CBB cycle increase significantly during the day, possibly to support higher flux through this cycle (Saha et al., 2016; Vijayan et al., 2009; Welkie et al., 2019). Our results suggest that the flux through the CBB cycle may also be affected by the metabolite levels, a hypothesis that needs to be tested in future studies.

While comparing these results with the reported absolute metabolite pool sizes in other cyanobacteria (Dempo et al., 2014), we note that metabolites with large absolute pool sizes such as glutamate and nucleotides such as ATP and ADP show smaller fold changes, whereas those that are present in smaller pool size such as RuBP, SBP, and FBP show large fold changes. Next, it was of interest to compare our results with a recent study on metabolome profiling of *Synechocystis* sp. PCC 6803, in the diurnal cycle (Werner et al., 2019). Importantly, data on the key CBB cycle intermediates such as SBP, S7P, RuBP, and 3PGA were absent in that study. These compounds show large fold changes in the present study. Other intermediates of the CBB cycle showed dampened oscillations in *Synechocystis* sp. 6803 compared with the present study (Werner et al., 2019). Another recent study reports much smaller oscillations in the levels of the CBB cycle metabolites in *Synechocystis* sp. 6803 in light-dark cycle when compared with other cyanobacteria (Will et al., 2019). Despite remarkable changes in the expression of genes of the CBB cycle in *Synechocystis* sp. 6803 under diurnal conditions (Saha et al., 2016), negligible change in metabolite levels may suggest a potential limitation of metabolic capacity in that strain. Notably, none of the previous studies of diurnal growth report the accumulation patterns for sugar nucleotides and γ-glutamyl peptides that show significant fold changes in the present study.

We have used the strategy of replicate sampling combined with the ion-pairing based, reverse-phase chromatography method that allows detection, identification, and quantification of a large number of intermediate metabolites. Furthermore, the use of isotopic area ratios with ^13^C-labeled IS improved the quality of our results by dramatically reducing the intra-sample CV (Figure S5). We chose time points based on the light regime and the instantaneous growth rates (Figure 2A). The midday and midnight samples were apparent choices, with the former also coinciding with the peak growth rate. The morning and evening points are characterized by light intensity of ~15% of the peak but negligible or even negative instantaneous growth rates (Figure 2A). The sample at CL provided an excellent comparison with that at midday and highlighted the key differences between growths under CL or LD regimes. Importantly, this strategy allowed us to detect statistically significant changes in an unprecedented number of metabolites. We believe that the present study will not only provide guidance for metabolic models but also become the basis for future studies that may involve multiple time points and additional metabolites, possibly detected via multiple analytical platforms.

### Limitations of the Study

First, we present only the relative changes in the metabolite levels and not their absolute concentrations. Absolute quantification poses three key challenges: (1) the need for pure standards, which are known to be expensive; (2) the loss of metabolites during extraction and storage; and (3) matrix effects in quantification. We offset the last two effects by using metabolite extracts of ^13^C-labeled biomass as IS. Second, we chose replicate sampling resulting in fewer time points in a diurnal cycle, and this may be considered as a limitation of the present study. In the absence of any resource limitation, it would be desirable to monitor a large number of metabolites at frequent intervals with many replicates and for two consecutive diurnal cycles. However, the experimental design needs to be considered in view of the objectives and limited resources, which in the present context was the LC-MS time. We aimed to achieve the following tasks with the available LC-MS time, notionally equivalent to 100 injections: (1) LC-MS method development, (2) metabolite identification, (3) testing of the ^13^C-labeled metabolite extract of cyanobacteria to serve as IS, and (4) performing the main experiment. Previous studies have mooted dense sampling strategies, with a small number of replicates, for time course experiments in biology with the rationale that they can re-create the values at non-sampled time points more accurately than replicate sampling (Sefer et al., 2016). The strategy assumes that the data inherently contain several transitions. A large number of proteomics and transcriptomics studies of cyanobacteria suggest to the contrary, thus categorizing most genes as dusk or dawn-peaking (Welkie et al., 2019). This supports our approach of collecting data for fewer time points based on the transitions in specific growth rate. Another limitation of our study is that we could not perform detailed investigations into the negative instantaneous growth rates observed during the dark to light and light to dark transitions and the other interesting observations in the growth profile. Further studies may be needed to first identify if these result from changes in the cell number or cell size followed by physiological studies involving oxygen evolution rates and quantum yield around these transitions. Finally, gene expression studies under diurnal cycles may provide a clearer picture on the pathways that may be more active during the dark phase.

### Resource Availability

#### Lead Contact

Requests for resources should be directed to the Lead Contact, Pramod P. Wangikar (wangikar@iitb.ac.in)

#### Materials Availability

This study did not generate new unique reagents.

#### Data and Code Availability

All data generated and analyzed in this study are included in this article and its Supplemental Information files. The raw data files for the metabolomics study of *Synechococcus elongatus* PCC 11801 under the diurnal cycle presented in this article are deposited to the Metabolomics Workbench repository (http://www.metabolomicsworkbench.org/), DOI: https://doi.org/10.21228/M8JM4K.

## Methods

All methods can be found in the accompanying Transparent Methods supplemental file.

## Supplemental Information

Document S1. Transparent Methods, Figures S1–S5, and Tables S1–S4

Table S5. List of 67 Metabolites Reported in the Study, Related to Figures 1, 2, and 3

Table S6. Area Ratios of 67 Targeted Metabolites, Related to Figure 3

Table S7. Area Ratios of 41 Unannotated m/z Features, Related to Figure 4

Table S8. Number of C and N Atoms for 41 Unannotated m/z Features, Related to Figure 4

## Footnotes

Supplemental Information can be found online at https://doi.org/10.1016/j.isci.2020.101704.
