# Supplementary material for: Dynamic Inventory of Intermediate Metabolites of Cyanobacteria in a Diurnal Cycle
Source: iScience. 2020 Oct 20;23(11):101704. doi: 10.1016/j.isci.2020.101704 (PMC7644974; doi:10.1016/j.isci.2020.101704)
Supplement: Document S1. Transparent Methods, Figures S1–S5, and Tables S1–S4 [file mmc1.pdf]

iScience, Volume *23*

Supplemental Information

Dynamic Inventory of Intermediate Metabolites of Cyanobacteria in a Diurnal Cycle

Damini Jaiswal and Pramod P. Wangikar

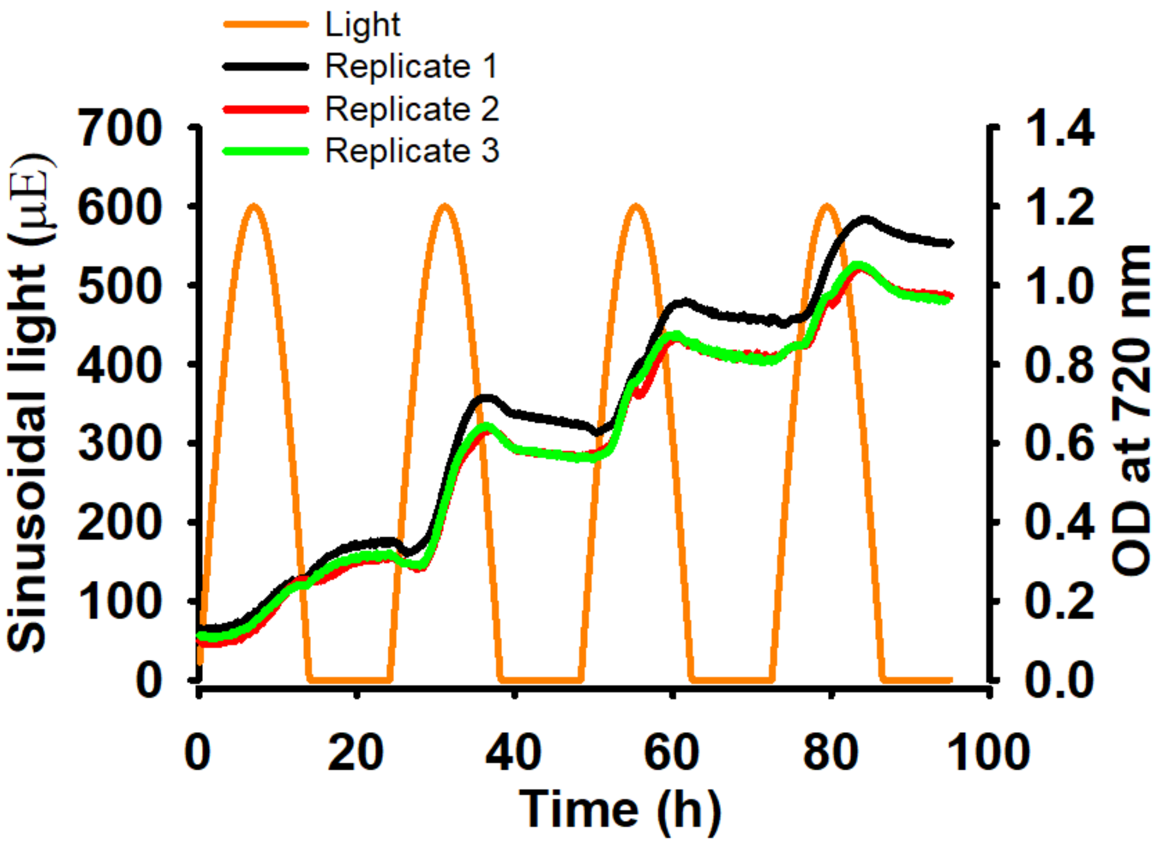

Figure S1: The growth profile of *S. elongatus* PCC 11801 under the diurnal condition at a light amplitude of 600 μmole photons.m^{-2}. s^{-1} is shown. Related to Figure 2.

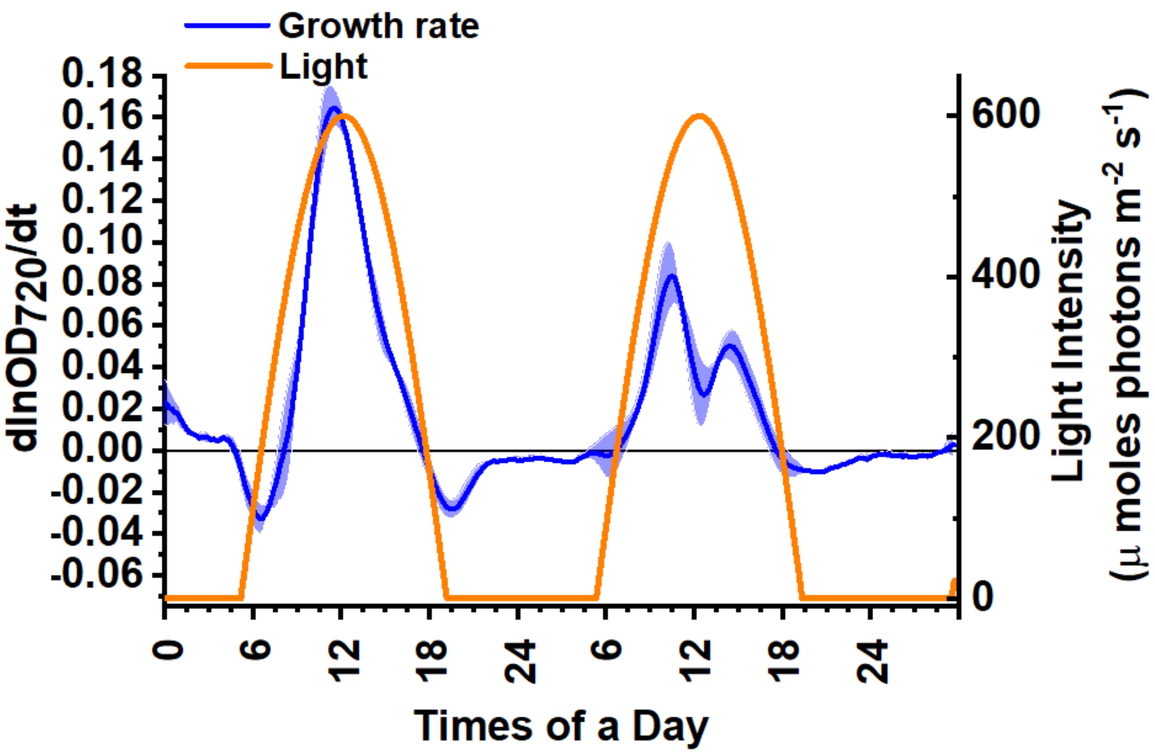

Figure S2: The light profile and instantaneous specific growth rate (μ) of two diurnal cycles (second and the third day) at a light amplitude of 600 μmole photons.m^{-2}. s^{-1} are shown. The samples were collected in the second diurnal cycle for metabolomics analysis. Related to Figure 2.

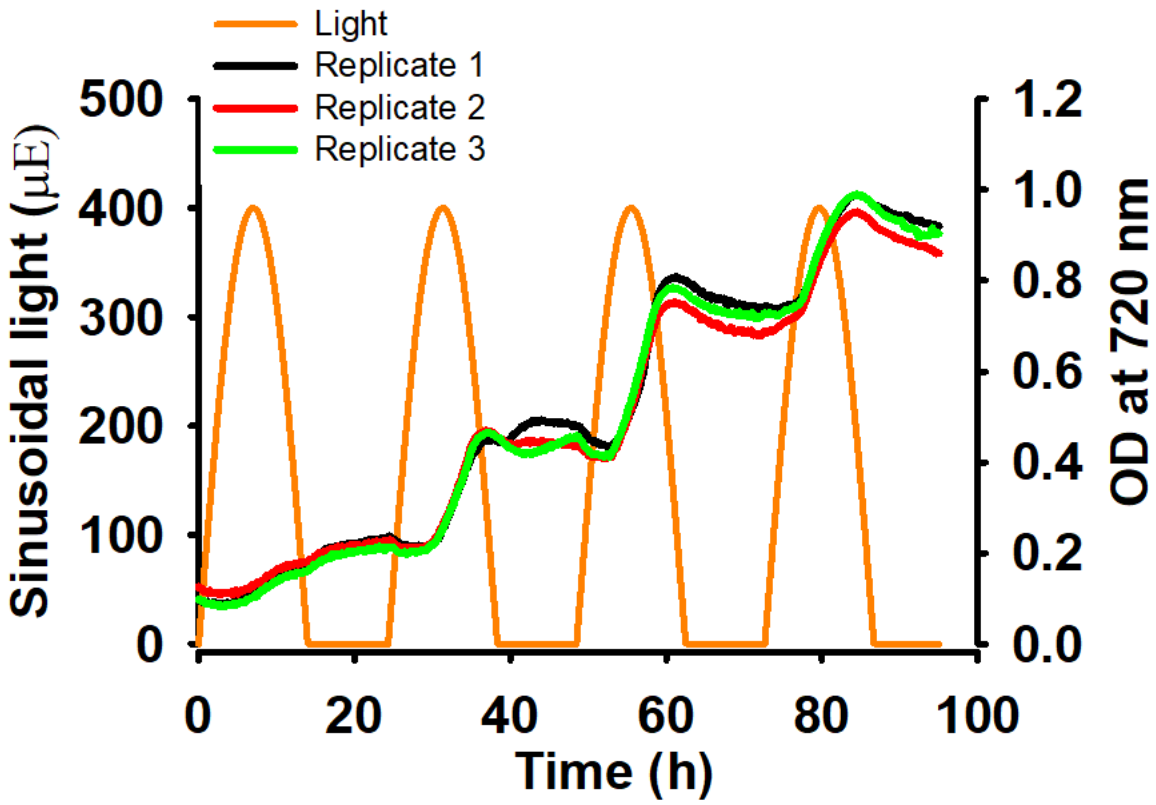

Figure S3: The growth profile of *S. elongatus* PCC 11801 under the diurnal condition at a light amplitude of 400 µmole photons.m^{-2}. s^{-1} is shown. Related to Figure 2.

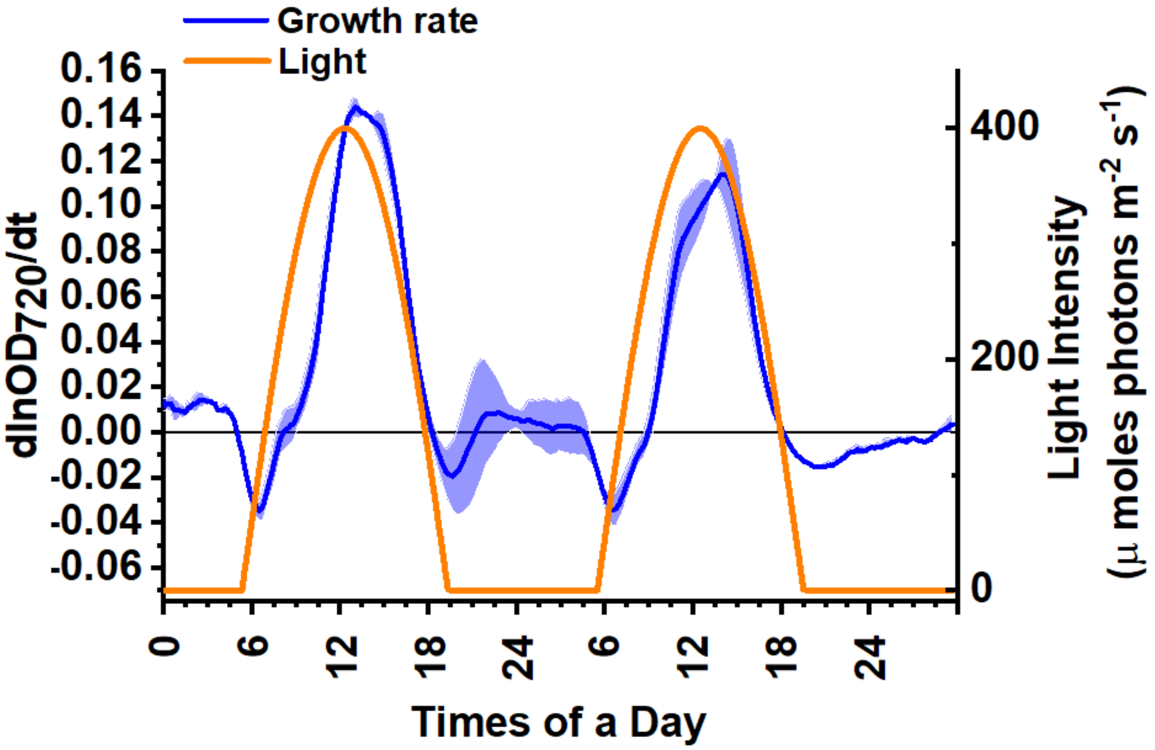

Figure S4: The light profile and instantaneous specific growth rate (μ) of two diurnal cycles (second and the third day) at a light amplitude of 400 μmole photons.m^{-2}. s^{-1} are shown. Related to Figure 2.

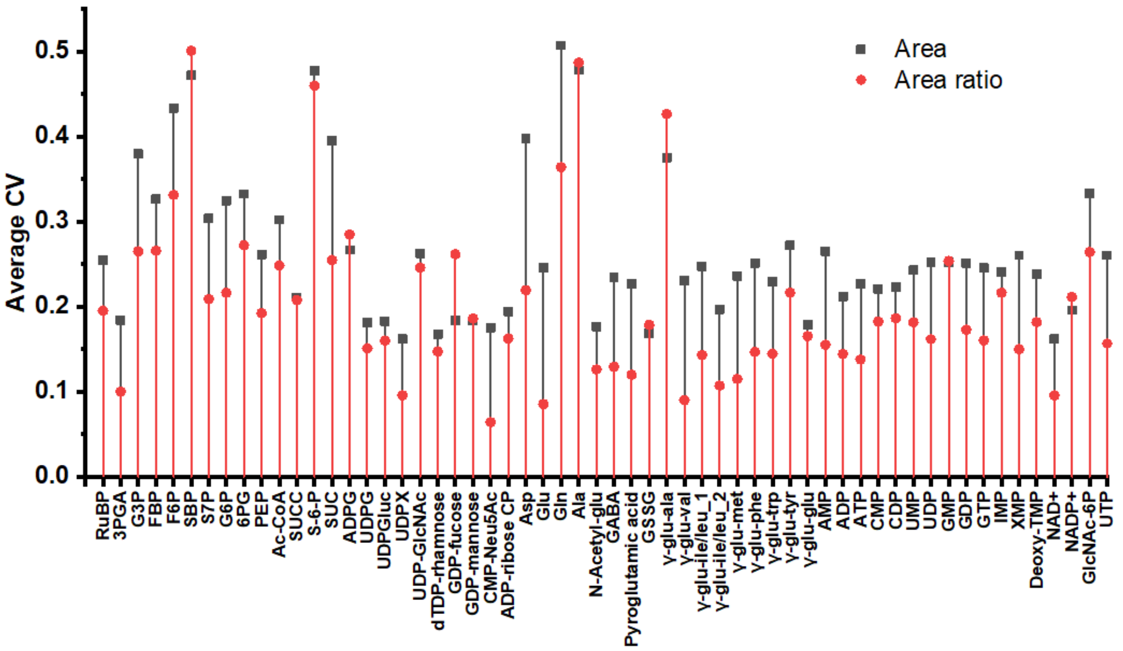

Figure S5: Comparison of the average coefficient of variation (CV) across the five conditions, morning (M), midday (MD), evening (E), midnight (MN), and continuous light (CL) when using peak areas and area ratios are shown. The CV values were lower when area ratios were used. Related to Figure 1.

Table S1: The parameters of ion source used for LCMS/MS data acquisition. Related to Figure 1.

**Source Parameters**	
Polarity	Negative
CUR	35 psi
GS1	40 psi
GS2	40 psi
Temperature	450 °C
ISVF	4500 V

SWATH Programs that were used to acquire the data for metabolite identification using MS-DIAL and MetDIA.

Table S2: SWATH Program 1 with a scan range of 80-680 m/z and Q1 width of 20 Da. The cycle time and accumulation time was 1.5 s and 47 ms, respectively. Related to Figure 1.

SWATH Exp	Start Mass	Stop Mass	CE	CE
SWATH Exp 1:	80	100	-13.8	15
SWATH Exp 2:	99	120	-14.9	15
SWATH Exp 3:	119	140	-16.1	15
SWATH Exp 4:	139	160	-17.2	15
SWATH Exp 5:	159	180	-18.4	15
SWATH Exp 6:	179	200	-19.5	15
SWATH Exp 7:	199	220	-20.7	15
SWATH Exp 8:	219	240	-21.8	15
SWATH Exp 9:	239	260	-23.8	15
SWATH Exp 10:	259	280	-24.1	15
SWATH Exp 11:	279	300	-25.3	15
SWATH Exp 12:	299	320	-26.4	15
SWATH Exp 13:	319	340	-27.8	15
SWATH Exp 14:	339	360	-28.7	15
SWATH Exp 15:	359	380	-29.9	15
SWATH Exp 16:	379	400	-31	15
SWATH Exp 17:	399	420	-32.2	15
SWATH Exp 18:	419	440	-33.3	15
SWATH Exp 19:	439	460	-34.5	15
SWATH Exp 20:	459	480	-35.6	15
SWATH Exp 21:	479	500	-36.8	15
SWATH Exp 22:	499	520	-37.9	15
SWATH Exp 23:	519	540	-39.1	15
SWATH Exp 24:	539	560	-40.2	15
SWATH Exp 25:	559	580	-41.4	15
SWATH Exp 26:	579	600	-42.5	15
SWATH Exp 27:	599	620	-43.7	15
SWATH Exp 28:	619	640	-44.8	15
SWATH Exp 29:	639	660	-46	15
SWATH Exp 30:	659	680	-47.1	15

Table S3: SWATH Program 2 with a scan range of 70-650 m/z and Q1 width of 20 Da. The cycle time and accumulation time was 1.5 s and 48 ms, respectively. Related to Figure 1.

SWATH Exp Index	Start Mass (Da)	Stop Mass (Da)	CE	CES
SWATH Exp 1:	70	90	-13.2	15
SWATH Exp 2:	89	110	-14.3	15
SWATH Exp 3:	109	130	-15.5	15
SWATH Exp 4:	129	150	-16.6	15
SWATH Exp 5:	149	170	-17.8	15
SWATH Exp 6:	169	190	-18.9	15
SWATH Exp 7:	189	210	-20.1	15
SWATH Exp 8:	209	230	-21.2	15
SWATH Exp 9:	229	250	-22.4	15
SWATH Exp 10:	249	270	-23.8	15
SWATH Exp 11:	269	290	-24.7	15
SWATH Exp 12:	289	310	-25.9	15
SWATH Exp 13:	309	330	-27	15
SWATH Exp 14:	329	350	-28.2	15
SWATH Exp 15:	349	370	-29.3	15
SWATH Exp 16:	369	390	-30.5	15
SWATH Exp 17:	389	410	-31.6	15
SWATH Exp 18:	409	430	-32	15
SWATH Exp 19:	429	450	-33.9	15
SWATH Exp 20:	449	470	-35.1	15
SWATH Exp 21:	469	490	-36.2	15
SWATH Exp 22:	489	510	-37.4	15
SWATH Exp 23:	509	530	-38.5	15
SWATH Exp 24:	529	550	-39.7	15
SWATH Exp 25:	549	570	-40.8	15
SWATH Exp 26:	569	590	-42	15
SWATH Exp 27:	589	610	-43.1	15
SWATH Exp 28:	609	630	-44.3	15
SWATH Exp 29:	629	650	-45.4	15

Table S4: SWATH Program 2 with a scan range of 458-950 m/z and Q1 width of 25 Da. The cycle time and accumulation time was 1.5 s and 70 ms, respectively. Related to Figure 1.

**SWATH Exp Index**	**Start Mass (Da)**	**Stop Mass (Da)**	**CE**	**CES**
SWATH Exp 1:	458	483	-35.6	15
SWATH Exp 2:	482	508	-37.0	15
SWATH Exp 3:	507	533	-38.4	15
SWATH Exp 4:	532	558	-39.8	15
SWATH Exp 5:	557	583	-41.3	15
SWATH Exp 6:	582	608	-42.7	15
SWATH Exp 7:	607	633	-44.2	15
SWATH Exp 8:	632	658	-45.6	15
SWATH Exp 9:	657	683	-47.0	15
SWATH Exp 10:	682	708	-48.5	15
SWATH Exp 11:	707	733	-49.9	15
SWATH Exp 12:	732	758	-51.3	15
SWATH Exp 13:	757	783	-52.8	15
SWATH Exp 14:	782	808	-54.2	15
SWATH Exp 15:	807	833	-55.7	15
SWATH Exp 16:	832	858	-57.1	15
SWATH Exp 17:	857	883	-58.5	15
SWATH Exp 18:	882	908	-60.0	15
SWATH Exp 19:	907	933	-61.4	15
SWATH Exp 20:	932	950	-62.8	15

Transparent Methods

EXPERIMENTAL MODEL AND SUBJECT DETAILS

Strain and Cultivation Conditions

The wild-type strain *S. elongatus* PCC 11801 isolated from Powai Lake, India, has been reported previously (Jaiswal *et al.*, 2018*b*). The strain can be accessed by requesting the lead contact or the Pasteur Culture Collection of Cyanobacteria (PCC). The strain was maintained in a shaker (New Brunswick Innova 44R, Eppendorf, Hamburg, Germany) at 38 °C under ambient air, 120 rpm, and a light intensity of ~350 µmole photons.m^{-2}. s^{-1} unless specified otherwise. BG-11 medium with an initial pH of 7.5 was used for cultivation. For metabolome profiling, the culture was grown in a multicultivator (Photon Systems Instruments, MC 1000-OD, Drasov, Czech Republic) under diurnal lighting; 14 h sinusoidal light peaking at 600 µmole photons.m^{-2}. s^{-1} and 10 h dark (Figure 2A) grown at 38 °C and bubbled with ambient air at ~ 1 volume/volume/min (vvm). Exponentially growing cultures (OD_{720} ~0.5-0.6) were used to inoculate for the LD cycle. The OD_{720nm} data was acquired every 10 min. The instantaneous specific growth rate (µ) during the diurnal cycle was calculated from the slope of the semi-logarithmic plot of OD_{720nm} versus time for eight consecutive time points. The cultures were also grown at a continuous light of 600 µmole photons.m^{-2}.s^{-1} under otherwise identical conditions.

METHOD DETAILS

Sampling and Extraction

The culture was inoculated at the diurnal time of 5:00 h on day 1 and sampled at 6:00, 12:00, 18:00, and 24:00 h on day 2, thus corresponding to the 25th, 31st, 37^{th,} and 43rd hour after inoculation. The sampling volume was adjusted to draw biomass equivalent to 12 OD_{720}.ml. The samples were fast-filtered through nylon membrane filters (Whatman, 0.8 µ, catalog no. 7408-004) in the presence of light that was roughly equivalent to the light-intensities in multi-cultivator at the time of sampling. The samples of cells growing at a continuous light intensity of 600 µmole photons.m^{-2}. s^{-1} was also collected at an OD_{720} ~0.6 for analysis. The filtered samples were rapidly quenched in 80/20 methanol-water (precooled at -80°C), and cells soaked in methanol were stored at -80°C for an hour. The cells were then removed off the filter using precooled chloroform (-20°C). The cells with methanol-chloroform mixture were vortexed for 25 minutes under cold conditions. Next, 0.2 M ammonium hydroxide solution was added and vortexed for 10 minutes. The sample was then centrifuged at 8000 g for 15 minutes. The aqueous layer was collected in two aliquots (≈ 1.5 mL each) and lyophilized. The lyophilized extracts were stored at -80°C until analyzed using LCMS. The concentration of methanol:chloroform:water was maintained as 1:2:1 during extraction. (Jaiswal *et al.*, 2018*a*; Prasannan *et al.*, 2018).

Preparation of ^{13}C-labeled biomass

A protocol was developed to grow *S. elongatus* PCC 11801 with $NaH^{13}CO3$ obtain metabolite extract that shows dominant ^{13}C monoisotopic peaks and no ^{12}C monoisotopic peaks for all metabolites(Jaiswal *et al.*, 2020*b*). A modified form of BG-11 medium that does not contain any organic carbon source such as sodium carbonate, citric acid, and ferric ammonium citrate (BG11-C^{-}) was used to prepare ^{13}C-enriched metabolite extracts of PCC 11801. Iron sulfate heptahydrate was used to replace ferric ammonium citrate. The exponentially growing culture of *S. elongatus* PCC 11801 pre-adapted to BG11-C^{-} medium was used for inoculation with an OD_{720} nm of 0.05 and a culture volume of 20 mL in 100 mL Erlenmeyer flask. The use of lower biomass for inoculation ensured minimal dilution with ^{12}C present in the biomass of the inoculum. The exchange of $^{12}CO_2$ from the environment was prevented using a stopper. ^{13}C-labeled sodium bicarbonate (^{13}C, 99 atom %) purchased from Cambridge Isotope Laboratories (Andover, MA, USA) was used as a substrate. The first addition of ^{13}C-labeled sodium bicarbonate was done at one hour of inoculation and a

concentration of 2 g/L. Subsequent doses of $NaH^{13}CO_3$ were provided at 18, 19.5, 21, and 22.5 hours after inoculation at a final concentration of 1 g/L. The entire procedure of ^{13}C-labeling was carried out in a shaker maintained at ambient CO_2, 38°C, 120 rpm, and a light intensity of 300 µmole photons.m^{-2}. s^{-1}. The ^{13}C-labeled biomass was harvested at 23 h by fast filtration in the presence of light followed by rapid quenching in 80/20 methanol-water (precooled to -80 °C). The ^{13}C-labeled metabolites were extracted from quenched cells, as described above. The ^{13}C-labeled metabolite extracts were filtered, and multiple aliquots of equal volume were dispensed in Eppendorf tubes. The extracts were lyophilized and stored at -80°C until ready for use.

^{15}N-Labeling Experiment

The ^{15}N-labeling was carried out in a shaker maintained at ambient CO_2, 38°C, 120 rpm, and a light intensity of 300 µmole photons.m^{-2}. s^{-1}. ^{15}N sodium nitrate (^{15}N, 98%) was purchased from Cambridge Isotope Laboratories (Andover, MA, USA) was used as a labeled substrate. An exponentially growing culture of *S. elongatus* PCC 11801 was used to inoculate in 20 mL BG-11 medium lacking sodium nitrate with an OD_{720} nm of 0.05. In this medium, ^{15}N-labeled sodium nitrate ($Na^{15}NO_3$) was added to a concentration equivalent to that of sodium nitrate present in the original BG-11 medium (1.5 g/L). The culture was allowed to accumulate the biomass up to an OD_{720nm} of 0.7. The cells were then filtered and extracted with a protocol described above.

Instrumentation, Sample Preparation, and Data Acquisition

A Triple TOF 5600+ mass spectrometer (Sciex, Framingham, MA) coupled to a Shimadzu ultra-performance-liquid chromatography (UPLC) system (Shimadzu, Nexera LC-30 AD, Singapore) equipped with a binary pump, degasser, column oven, and autosampler was used for the analysis. The instrument was operated in negative ion mode (details of ion source parameters can be found in Table S1). To quantify the isotopic ratios of the metabolites, LCMS data was acquired using the information-dependent acquisition method (IDA) and was set up to collect MS/MS information for the top 10 peaks with an m/z range of 50-1000 Da. The metabolite extracts from each condition were reconstituted in 100 µL of 50:50 methanol-water and filtered using nylon syringe filters to remove any particulate matter. An equal volume of ^{13}C labeled internal standard was added to all the samples. The injection volume was 6µL. Chromatographic separation was achieved using reverse-phase ion-pairing chromatography on C18 Synergi 4 µm Hydro-RP LC column 150 x 2 mm (Phenomenex Inc, Torrance, CA) using a gradient elution method consisting of eluents, 10 mM tributylamine + 15mM acetic acid in water (pH = 4.95) (buffer A) and 100% Methanol (buffer B) (Qian *et al.*, 2004; Luo *et al.*, 2007; Lu *et al.*, 2010; Mccloskey and Gangoiti, 2015). The gradient method used is as follows: 0% B (0.01 min), 0% B (2 min), 35% B (8 min), 35% B (10.5 min),90% B (15.50 min), 90% B (20.5 min), 0% B (22 min), and 0% B(30 min)(Jaiswal *et al.*, 2020*a*).

Additionally, the data-independent acquisition (DIA) method of SWATH was also employed on the above chromatography with the objective to improve the coverage of the identified metabolites. SWATH acquires MS2 data for user-defined Q1 isolation windows. To ensure that metabolites of the entire m/z range get covered and to keep the cycle time under 2 s, we designed three SWATH-MS programs. These included two programs with shifted windows and one program for a higher molecular weight range (see Table S2-S4 for details of the SWATH programs).

QUANTIFICATION AND STATISTICAL ANALYSIS

Metabolite Identification and Verification

The metabolites reported in this study were identified using MS-DIAL(version 3.30) and MetDIA (version 1.03) tools at the MS2 level (Tsugawa *et al.*, 2015; Li *et al.*, 2016). The .wiff files generated by the instrument were converted to .abf and .mzML format using abf converter (https://www.reifycs.com/AbfConverter/) and proteowizard MS convert tool

(http://proteowizard.sourceforge.net/tools.shtml) for use in MS-DIAL and MetDIA, respectively. For metabolite identification using MS-DIAL, MSMS-AllPublic-Curated-Neg library available at PRIMe (http://prime.psc.riken.jp/) was used. In the case of MetDIA, the library provided with the package was used. The mass tolerance at MS1 and MS2 level were kept at 25 and 35 ppm, respectively, and a score cut off of 0.8 was used for library matching. Further, over 50% of the central metabolites were confirmed by injecting pure standards (Table S5). The structural annotation of metabolites identified through MS-DIAL and MetDIA was performed using respective .mol file obtained from the Kyoto Encyclopedia of Genes and Genomes (KEGG)(Kanehisa and Goto, 2000) or Human Metabolome Database (HMDB)(Wishart *et al.*, 2007) using inbuilt fitting algorithm running under PeakView 2. Environment 2 (Sciex, Framingham, MA). The chemical formula of metabolites and their fragments was verified by monitoring the mass shifts in the fully labeled ^{13}C and ^{15}N labeled metabolite samples of *S.elongatus* PCC 11801 using PeakView2.2 and Master View 1.0 (Sciex, Framingham, MA). The C and N composition of the unannotated m/z features was obtained from the mass shifts in the respective ^{13}C or ^{15}N labeled samples using the X^{13}CMS tool (Huang *et al.*, 2014).

Data Processing

The extracted ion chromatograms (XIC) of precursor metabolites were visualized using PeakView2.2 and MasterView 1.0 (Sciex, Framingham, MA). Quantification of the peak areas of the annotated metabolites listed in Figures 2, 3 and Table S5 was performed using MultiQuant 3.0.1 (Sciex, Framingham, MA). Quantification of the peaks of the unannotated m/z features was obtained by submitting a multigroup job on the XCMS Online portal (Tautenhahn *et al.*, 2012; Li *et al.*, 2013). Ratios of the ^{12}C and corresponding ^{13}C monoisotopic peaks were used for the relative quantification of metabolites. The area ratios for all the metabolites in each condition presented in the study have been provided as Table S6 and S7.

Statistical Analysis

All the experiments were performed in three biological replicates with three replicate LCMS injections for each sample. Isotopic area ratios were first estimated for each replicate, $\log_2$ transformed, averaged over the replicates, and autoscaled to plot heatmaps using OriginPro software (version 9.6.5.169). The maximum fold-change across the five conditions for each metabolite was obtained by dividing the highest average area ratio by the lowest one. The error of fold-change between two conditions was calculated using the Taylor expansion method according to the following equation (Wolter, 2007):

$$\sigma^2{}_{\frac{X}{Y}} = \frac{1}{\bar{\bar{Y}}^2}\sigma_X^2 + \frac{\bar{X}^2}{\bar{Y}^4}\,\sigma_Y^2 - 2\frac{\bar{X}}{\bar{Y}^3}\,cov\,(X,Y)$$

where σ denotes the variance, X and Y denote two conditions, $\bar{X}$ and $\bar{Y}$ are the means of the area ratios for a metabolite in the conditions X and Y, respectively, and cov (X, Y) denotes the sample covariance. The fold change across two conditions are represented as:

$$\frac{\bar{X}}{\bar{Y}} \pm \frac{\sigma_{\frac{X}{Y}}}{\sqrt{n}}$$

where n denotes the number of replicate measurements. The principal component analysis was performed using MetaboAnalyst 4.0(Chong *et al.*, 2018). ANOVA was performed to measure the significance of the four LD cycle conditions. Student's t-test was performed to compare across two conditions presented in this study. The targeted set of metabolites that showed a fold change of ≥1.5 and ≤0.66 and a p-value of ≤0.05 were considered as significant. For unannotated m/z features, an additional criterion of the coefficient of variation (CV) less than 0.3 was used.

Supplemental References

Chong J, Soufan O, Li C, Caraus I, Li S, Bourque G, Wishart DS, Xia J. 2018. MetaboAnalyst 4.0: Towards more transparent and integrative metabolomics analysis. Nucleic Acids Research **46**, W486–W494.

Huang X, Chen YJ, Cho K, Nikolskiy I, Crawford PA, Patti GJ. 2014. X13CMS: Global tracking of isotopic labels in untargeted metabolomics. Analytical Chemistry **86**, 1632–1639.

Kanehisa M, Goto S. 2000. KEGG: kyoto encyclopedia of genes and genomes. Nucleic acids research **28**, 27–30.

Li S, Park Y, Duraisingham S, Strobel FH, Khan N, Soltow QA, Jones DP, Pulendran B. 2013. Predicting Network Activity from High Throughput Metabolomics. PLoS Computational Biology **9**.

Prasannan CB, Jaiswal D, Davis R, Wangikar PP. 2018. An improved method for extraction of polar and charged metabolites from cyanobacteria (F Chauvat, Ed.). PLOS ONE **13**, e0204273.

Tautenhahn R, Patti GJ, Rinehart D, Siuzdak G. 2012. XCMS Online: a web-based platform to process untargeted metabolomic data. Analytical Chemistry **84**, 5035–5039.

Wishart DS, Tzur D, Knox C, *et al.* 2007. HMDB: The human metabolome database. Nucleic Acids Research **35**, 521–526.

Wolter KM. 2007. Taylor Series Methods. Introduction to Variance Estimation. Springer New York, 226–271.
